# Expression of concern: ‘Involvement of miR-4262 in paclitaxel resistance through the regulation of PTEN in non-small cell lung cancer’ (2019), by Sun H *et al.*

**DOI:** 10.1098/rsob.230301

**Published:** 2023-08-31

**Authors:** Open Biology editorial team

Following the publication of ‘Involvement of miR-4262 in paclitaxel resistance through the regulation of PTEN in non-small cell lung cancer’, the Editorial team have concerns regarding the validity of the data used in this study.

We are issuing an expression of concern here and investigating this as a matter of urgency; we will notify readers as to the results of our investigation as soon as possible.

